# Exploring User Behavior, Profiles, and Generation of Missed Reading Alerts in Long-Term Users of a Technology-Enabled Intervention for Self-Monitoring of Blood Pressure in Public Primary Care Setting in Singapore: Longitudinal Observational Study

**DOI:** 10.2196/74051

**Published:** 2025-09-22

**Authors:** Shilpa Tyagi, Keith Chiaw Meng Sng, David Wei Liang Ng, Valerie Hui Ying Teo, Chun Yen Beh, Evon Oh, Jeremy Cong En He, Scott Joel Yu Jie Heng, Gerald Choon-Huat Koh

**Affiliations:** 1 MOH Office for Healthcare Transformation (MOHT), 1 North Buona Vista Link, #09-02, Singapore, 139691, Singapore, 65 91824203; 2 Saw Swee Hock School of Public Health, National University of Singapore, Singapore, Singapore; 3 National Healthcare Group Polyclinics (NHGP), Singapore, Singapore; 4 National University Polyclinics (NUP), Singapore, Singapore; 5 SingHealth Polyclinics, Singapore, Singapore

**Keywords:** telemonitoring, user engagement, hypertension, user profile, user attrition, remote monitoring, user interaction, telecare, eHealth

## Abstract

**Background:**

Technology-enabled interventions for chronic disease management, such as telehealth systems for hypertension self-monitoring, have demonstrated effectiveness but face challenges with sustained usage and high attrition rates. Understanding the factors associated with continued engagement is crucial for enhancing intervention design and sustainability.

**Objective:**

This study aimed to explore the user behavior and user profiles under the Primary Technology Enhanced Care for Hypertension Program (PTEC-HT) intervention by: (1) quantitatively describing characteristics of participants generating Missed Reading (MR) alerts, (2) identifying factors associated with MR alert generation, (3) profiling participant subgroups based on MR alert patterns and blood pressure (BP) control, and (4) examining temporal trajectories of MR alerts and associated conversion rates over 12 months.

**Methods:**

A longitudinal observational study was conducted using backend data from the PTEC-HT system. The study included 491 participants, recruited before June 30, 2022, enrolled in the program for 1 year or more, categorized into MR alert generator and nongenerator groups, recruited before June 2022. Logistic regression identified factors associated with MR alert generation in an index month (August 2023), while latent class analysis profiled participant subgroups. Generalized estimating equations examined temporal trajectories of MR alerts and conversion rates. Statistical significance was set at 5%.

**Results:**

Being younger (odds ratio [OR] 0.97, 95% CI 0.95‐0.99; *P*=.007) and having a longer program duration (OR 1.11, 95% CI 1.01‐1.22; *P*=.03) were significantly associated with MR alert generation. Latent class analysis identified 3 latent classes: (1) Compliant Triers (low MR alerts, poor BP control; 56/491, 11.4%), (2) Compliant Achievers (low MR alerts, good BP control; 368/491, 74.9%), and (3) Non-Compliant Achievers (high MR alerts, good BP control; 67/491, 13.6%). Temporal analysis showed consistent trajectories for Missed Reading Reminder message counts and conversion rates, with MR alert generators having higher Missed Reading Reminder message counts but lower conversion rates compared to nongenerators.

**Conclusions:**

Our study reported that younger participants and longer program durations were linked to higher MR alert generation. The identification of distinct user profiles suggests that tailored intervention features could enhance engagement and BP control. The study underscores the importance of monitoring compliance patterns and optimizing message content to improve conversion rates. These insights contribute to the understanding of telehealth engagement dynamics and support targeted interventions for hypertension management.

## Introduction

### Overview

While there is evidence to support that interventions involving self-management of chronic diseases by patient empowerment are effective [1-3], with an increasingly used modality being telehealth interventions, the flip side of telehealth interventions is the large attrition rate associated with time. It has been reported in literature that up to 80% of users of mobile apps often use such interventions minimally with high rates of dropout. Among the included studies, the time period of app usage varied from 6 months to up to 23 months in a systematic review conducted by Fleming and colleagues [4], while the app usage period was reported as 2 years by Pfammatter and colleagues [5]. In addition, another observational study situated in a real-world setting reported less than 5% of the participants having continuous usage of the mobile app [6]. A recent systematic review synthesizing quantitatively and qualitatively the evidence on attrition associated with mobile health interventions reported the dropout rate to be high (40% for 9 randomized controlled trials and 49% for 8 observational studies with an overall pooled rate of 43%). The time period of app usage in these included studies varied from 4 weeks to up to 1 year. The reasons cited were social, demographic, and behavioral which were classified as modifiable [7]. This not only highlights the magnitude of the attrition problem but also provides a sound basis for studying the experience of sustained usage of technology-enabled interventions for self-management of chronic disease and the associated factors. This study would address this gap with the use case of patients with hypertension within the Primary Technology Enhanced Care for Hypertension Program (PTEC-HT) in Singapore [8].

PTEC-HT was developed to integrate telehealth solutions for hypertension management, emphasizing remote blood pressure (BP) monitoring, care team support through remote monitoring and follow-up teleconsultations, and medication adjustments based on clinical guidelines. Its evidence-based design incorporated features such as automated feedback, patient reminders, data visualization, and educational resources, ensuring practical and effective implementation. The Ministry of Health Office for Healthcare Transformation (MOHT), an innovation-focused unit aimed at health care redesign in Singapore [9], evaluated the program’s effectiveness through a quasi-experimental pilot study [10]. Building on the pilot’s success, MOHT has scaled PTEC-HT across the national public primary care system with the overall program duration being 5 years. This landmark initiative in telehealth for hypertension presents an opportunity to examine patient adherence to BP self-monitoring and sustained engagement with the system.

Within PTEC-HT, the key behavior that patients are expected to undertake is regular weekly submission of their BP readings into the PTEC-HT app. Based on the structure of the PTEC-HT system including in-app prompts and reminders, if the patient fails to submit the indicated BP reading within 1 week, they receive in-app Missed Reading (MR) reminders (a total of 2 per MR) to remind them to complete this task. However, the patient may choose to ignore these prompts, and after 4 consecutive weeks of no submitted readings, the care team in public primary care clinics gets an MR alert on the PTEC-HT clinician dashboard with predecided follow-up actions, most common being calling the patient to find out the reason for the MR alert. In a real-world setting, with increasing patient volume on PTEC-HT, not only is the study of this dimension of how patients interact with the PTEC-HT system important from a research and user-centered design perspective, but also from a program sustainability perspective to manage the care team’s workload. Hence, PTEC-HT provides an excellent opportunity to learn about the sustained use of a telemonitoring and telesupport intervention by patients with hypertension within a real-world implementation context as well as explore the patient interaction with the PTEC-HT system. The insights gained will not only help with the implementation and sustainability of the program but also add new knowledge to the existing literature.

### Aims and Objectives

The overall aim of this study was to assess user behavior, user profiles, the generation of MR alerts and associated factors in long-term users of a technology-enabled intervention (ie, PTEC-HT) in public primary care settings in Singapore. The following are the specific objectives:

1. To quantitatively describe the characteristics of a subgroup of PTEC-HT participants who contribute to MR alerts on the clinician dashboard based on the generation of MR alerts during the index month
2. To determine the factors associated with the generation of MR alerts during the index month in patients on PTEC-HT
3. To explore and quantify different profiles of PTEC-HT participants generating MR alerts during the index month, and to describe the characteristics of these identified profiles
4. To describe the temporal trajectories of generating MR reminders as well as the associated conversion rates by PTEC-HT patients over 12 months preceding the generation of MR alerts during the index month

## Methods

### Study Design

A longitudinal observational study design was adopted to address the above-stated objectives.

### Setting

**Figure 1.**
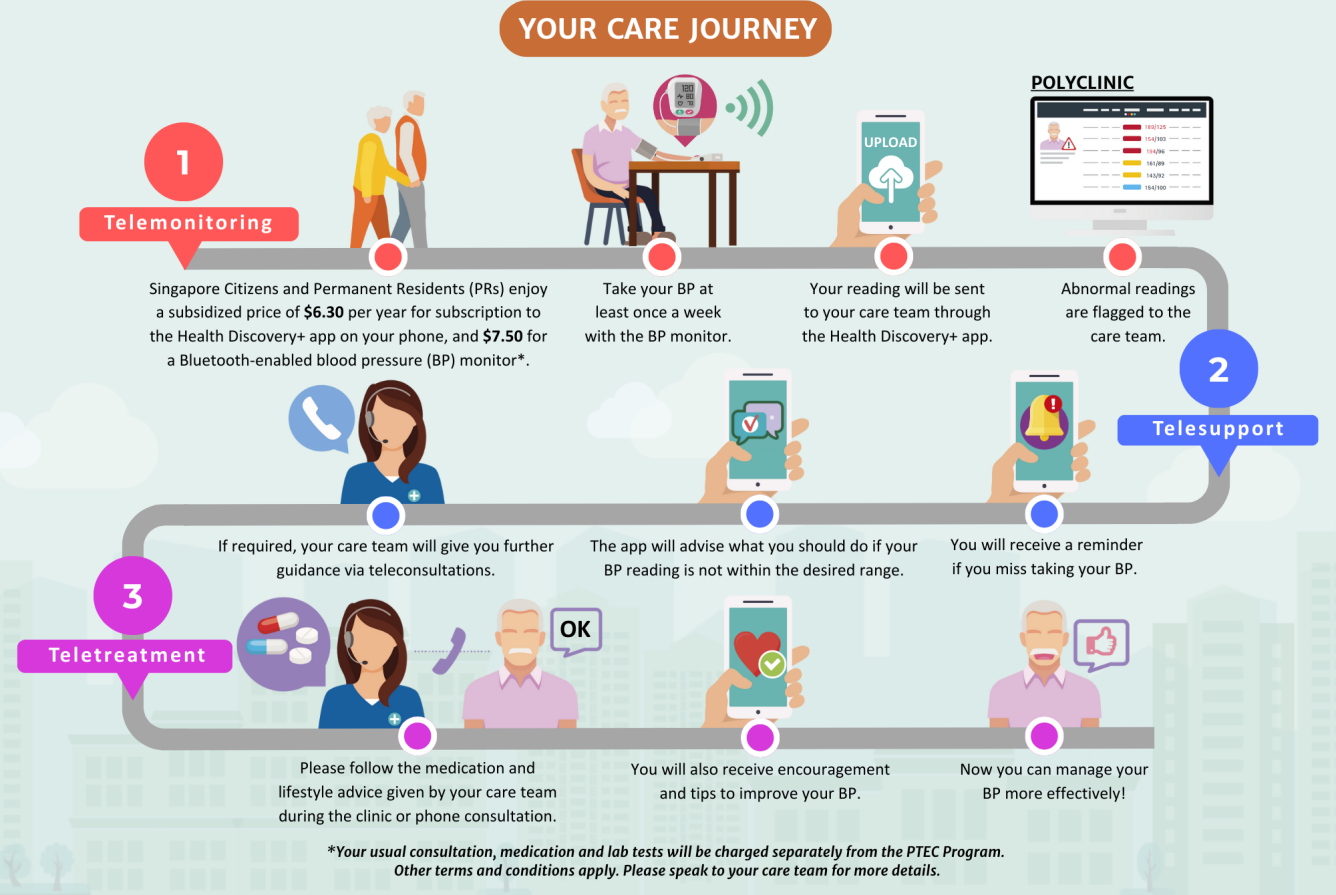
Patient care workflow or journey within the Primary Technology Enhanced Care for Hypertension scaling program. Source: PTEC-HT program team from MOHT. BP: blood pressure; PTEC: Primary Technology Enhanced Care Program.

The PTEC-HT is the first nationwide initiative under the Primary Technology Enhanced Care framework. It leverages simple, user-friendly technology and operates on the national IT platform, Vital Signs Monitoring (VSM), developed by Synapxe, Singapore’s health technology agency [8]. The program comprises 3 essential components designed to streamline care and improve patient outcomes. First, patients remotely monitor their BP using a Bluetooth-enabled BP machine, recording readings at least once a week. These readings are automatically transmitted to their public primary care clinic via the PTEC-HT app. Second, the care team provides regular support by reviewing the transmitted data. If a patient’s condition is poorly controlled or requires medication adjustments, the team initiates teleconsultations to provide timely interventions. Third, in-app assistance offers online support through advice, reminders, and a chatbot, helping patients stay engaged and adhere to their care plan [8]. The workflow or care journey for patients participating in PTEC-HT is illustrated in Figure 1, highlighting the seamless integration of technology and health care support to promote better management of high BP. At recruitment, participants are offered the choice of a default program duration of 1 or 2 years, after which the program is renewed regularly based on participant preferences. Implementation sites represented the 3 clusters of public primary care clinics within Singapore across which the program was implemented.

### Participants

Patients with hypertension are eligible to participate in PTEC-HT if they are aged between 21 and 80 years, not pregnant at the point of recruitment, have a history of high BP, and have no history of atrial fibrillation, kidney failure, or heart failure. The study population comprised “MR alert generator Group” and “non-MR alert generator Group.” While the “MR alert generator Group” comprised PTEC-HT participants who contributed to the generation of an MR alert in the month of August 2023 (ie, index period), the “non-MR alert generator Group” comprised PTEC-HT participants who did not contribute to the generation of an MR alert in the month of August 2023. For participants in both groups to be eligible, the PTEC-HT recruitment was anytime in June 2022 or before and no program termination between June 1, 2022, to July 31, 2023.

### Data Sources

The required data for this study were extracted from the PTEC-HT system backend. The operationalization of different time points for this data extraction is provided in Multimedia Appendix 1. The data extraction was structured around a defined timeline using the PTEC-HT system. For each eligible patient, the observation period was set as the 12 months prior to the contributing period (ie, July 1, 2022, to June 30, 2023). For the latent class analysis (LCA) (participant profiles), we used data from a 6-month segment (ie, January 1, 2022 to June 30, 2023) of that observation period, whereas for the temporal trajectory analysis of reminders and conversions, we used data from the entire 12-month observation period (ie, July 1, 2022 to June 30, 2023). The contributing period (July 1 to July 31, 2023) captured the 4-week span of nonsubmission of readings, and the index period (August 1 to August 31, 2023) marked when the MR alert outcome was assessed.

### Assessments

The main outcome of interest was the generation of an MR alert (ie, yes or no) during the index month. MR alert is generated on the clinician’s PTEC-HT dashboard if a participant did not submit any BP readings in the preceding month. This information was extracted for the designated index month of August 2023. The covariates included in the current analysis were as follows: age at recruitment, gender (ie, male or female), implementation site (ie, A, B, and C), duration on PTEC-HT, and baseline BP control. The duration on PTEC-HT was computed based on the PTEC-HT recruitment date and the index month over which the MR alert generator status was confirmed. Baseline BP control was defined based on the first BP reading submitted at the point of recruitment. A participant was categorized as having baseline BP control if they had both systolic BP<140 mm Hg and diastolic BP<90 mm Hg.

For the generation of PTEC-HT participant profiles, MR alert generation (ie, yes or no), BP control status, and monthly submission frequency of BP readings were computed over the preceding 6 months from the contributing period for MR alert generation. The cut-offs chosen for monthly BP control status were the same as those for baseline BP control. For assessing the temporal trajectories of generating MR reminders as well as the associated conversion rates over 12 months preceding the generation of MR alert during the index month, the following variables were extracted: Missed Reading Reminder A (MRRA), conversion to MRRA, Missed Reading Reminder B (MRRB), and conversion to MRRB. A participant would receive an MRRA if they did not submit any BP readings by the end of the week. Such a message is triggered by the PTEC-HT system every Saturday at 7 PM. Conversion to MRRA was categorized as yes if the participant submitted a BP reading after receiving the MRRA message and before the subsequent Monday, 9 AM. Conversion rate to MRRA was calculated as the proportion of the total number of conversions (coded as yes) to the total number of MRRA messages received in a month. A participant would receive an MRRB if they did not submit any BP readings over the week. Such a message is triggered by the PTEC-HT system on every Monday at 9 AM. Conversion to MRRB was categorized as yes if the participant submitted a BP reading after receiving the MRRB message and before the subsequent Wednesday. The conversion rate to MRRB was calculated as the proportion of the total number of conversions (coded as yes) to the total number of MRRB messages received in a month.

### Data Analysis

Descriptive characteristics were summarized using mean and SD for continuous variables, number and proportion for categorical variables. The bivariate association between the MR alert generator versus the nongenerator and predetermined covariates was assessed using a 2-tailed *t* test for continuous variables and chi-square test for categorical variables. Factors associated with the generation of MR alert during the index month were assessed by running logistic regression [11] with the outcome variable being MR alert generation during the index month (ie, yes or no). Chosen based on clinical relevance, prior evidence, and data availability, the following independent variables were included: age, gender, implementation site, baseline BP control, and program duration. These independent variables were organized by the block-wise modeling approach (Model I included sociodemographics, Model II added baseline BP control, and Model III added program duration) in a predetermined, staged manner. No automated stepwise selection (forward or backward) was used. Odds ratio (OR) along with 95% CIs for all 3 models are presented in the Results section.

To describe the profiles of PTEC-HT participants, LCA was conducted. LCA is a statistical method used to identify unobserved subgroups (latent classes) within a population based on observed data [12]. To identify latent classes, variables related to monthly MR alert generation, monthly BP control status, and monthly compliance with BP submission frequency over 6 months were included. Models with 2 to 3 classes were tested along with different combinations of the above 3 categories of variables. Three-class solution comprising MR alert generation and monthly BP control status variables was selected as the best fit based on statistical indicators of Akaike information criteria (AIC), Bayesian information criterion (BIC) [13] and interpretability of classes (refer to Multimedia Appendix 2).

The generalized estimating equation approach was used to analyze the temporal trajectories of generating MRRA, conversion rate to MRRA, MRRB, and conversion rate to MRRB over 12 months preceding the generation of the MR alert. Liang and Zeger [14] proposed the generalized estimating equation method for analyzing repeated measures data within the framework of generalized linear models, offering estimates that reflect population averages. While a Poisson distribution with a log link function was used for MRRA and MRRB data, a binomial distribution with a logit link was used for conversion to MRRA and conversion to MRRB data. We used a working correlation matrix based on an exchangeable correlation structure and used the Huber-White sandwich estimator to derive robust variance estimates, ensuring reliability even in cases where the working correlation matrix was incorrectly specified [14-16]. A simple model was run initially to obtain the unadjusted trajectories for MRRA, MRRB, conversion to MRRA, and conversion to MRRB with time (in months) as the independent variable (referred to as Model A). Model A was subsequently adjusted for age, gender, implementation site, baseline BP control, and duration of PTEC-HT (referred to as Model B). Subsequently, MR alert generation (during index month) was added to Model B (referred to as Model C). With this Model C, we further added interaction terms between the time variable and the MR alert generation variable to determine if the temporal trajectories vary by MR alert generation status (referred to as Model D). Sample size calculation was not conducted since data for all eligible participants were extracted. Significance level was set at 5%. All analyses were performed using Stata/SE 17 (StataCorp LLC) [17].

### Ethical Considerations

This study was reviewed and approved by the National University of Singapore’s Institutional Review Board (NUS-IRB Reference Code: NUS-IRB-2024‐1149). All participants provided informed consent upon enrollment in the PTEC-HT program, which covered the use of their data for research purposes. All extracted data were deidentified prior to analysis to protect privacy and confidentiality. Participants received no monetary compensation for participation in the program or the study.

## Results

A total of 491 patient participants from the PTEC-HT program, who met the inclusion criteria, were included in the current analysis. The descriptive characteristics comparing MR alert generators with non-MR alert generators are given in Table 1.

**Table 1.** Descriptive characteristics of Missed Reading Alert generator (during index month) versus not (N=491).

Characteristics	MR alert in index month (Yes) (n=93, 18.9%)	MR alert in index month (No) (n=398, 81.1%)	Total (N=491, 100%)	*P* value
Age (years), mean (SD)	58.6 (10.47)	61.6 (9.97)	61.0 (10.1)	.01
Age (years), n (%)				.14
65 years or less	64 (68.8)	241 (60.6)	305 (62.1)	
More than 65 years	29 (31.2)	157 (39.4)	186 (37.9)	
Gender, n (%)				.21
Female	44 (47.3)	160 (40.2)	204 (41.5)	
Male	49 (52.7)	238 (59.8)	287 (58.5)	
Implementation site, n (%)				.21
A	14 (15.1)	93 (23.4)	107 (21.8)	
B	63 (67.7)	240 (60.3)	303 (61.7)	
C	16 (17.2)	65 (16.3)	81 (16.5)	
Duration on PTEC-HT^a^ Program (months)^b^, mean (SD**)**	15.6 (2.3)	15.0 (2.3)	15.1 (2.4)	.04
Baseline BP control^c^, n (%)				.84
No	54 (58.1)	235 (59.2)	289 (59)	
Yes	39 (41.9)	162 (40.8)	201 (41)	

a PTEC-HT: Primary Technology Enhanced Care for Hypertension Program.

b Duration on the PTEC-HT Program is computed based on the time between the recruitment date for a patient participant and the start of the index month during which the Missing Reading alert generator status was confirmed.

c Baseline BP control is defined based on the first blood pressure reading submitted at the point of recruitment. A patient participant is categorized as having controlled baseline BP if they have both systolic BP<140 mm Hg and diastolic BP<90 mm Hg.

MR alert generators (mean 58.6, SD 10.5 years) were significantly younger than non-MR alert generators (mean 61.6, SD 9.97 years, *P*=.01). In addition, there was a statistically significant difference (*P*=.04) between both groups for duration on PTEC-HT program with MR alert generators having a longer duration (mean 15.6, SD 2.3 months) as compared to non-MR alert generators (mean 15, SD 2.3 months). There was no statistically significant difference between both groups for gender, implementation site, and baseline BP control. Referring to the adjusted analysis exploring the factors associated with the generation of MR alert during the index month in Table 2, the age of the participant as well as the duration on the PTEC-HT program were significantly associated with the generation of MR alert during the index month.

**Table 2.** Factors associated with the generation of the Missed Reading alert (during the index month) (N=491).

Characteristics	Model I^a^		Model II^b^		Model III^c^	
	OR^d^ (95% CI)	*P* value	OR^d^ (95% CI)	*P* value	OR^d^ (95% CI)	*P* value
Age (years)	0.97 (0.95‐0.99)	.008	0.97 (0.95‐0.99)	.009	0.97 (0.95‐0.99)	.007
Gender with reference to female (Male)	0.76 (0.48‐1.21)	.25	0.76 (0.48‐1.21)	.25	0.74 (0.46‐1.17)	.20
Implementation site with reference to A		.15		.16		.16
B	1.84 (0.98‐3.47)		1.83 (0.97‐3.46)		1.76 (0.93‐3.35)	
C	1.40 (0.63‐3.11)		1.39 (0.62‐3.10)		1.20 (0.53‐2.72)	
Baseline BP^h^ control with reference to No (Yes)	—^f^	—	0.98 (0.61‐1.57)	.94	1.03 (0.64‐1.66)	.89
Duration on PTEC-HT^g^ Program in months	—	—	—	—	1.11 (1.01‐1.22)	.03

a Model I: includes age, gender, and implementation cluster.

b Model II: Model I and baseline BP control status.

c Model III: Model II and duration on PTEC-HT program.

d OR: odds ratio.

e BP: blood pressure.

f Not available.

g PTEC-HT: Primary Technology Enhanced Care for Hypertension Program.

Specifically, older participants had lower odds of generating an MR alert as compared to younger participants (OR 0.97, 95% CI 0.95‐0.99; *P*=.007). For every additional month on the PTEC-HT program, the odds of generating an MR alert increased by 11% (OR 1.11, 95% CI 1.01‐1.22; *P*=.03).

While latent class membership and estimated probabilities of each observed variable are presented in Table 3, latent class size as well as key characteristics of each latent class are presented in Table 4. The following 3 latent classes were identified based on prevalence of MR alert generation as well as BP control status over 12 months: latent class 1 or “Compliant Triers,” latent class 2 or “Compliant Achievers,” and latent class 3 or “Non-Compliant Achievers.” “Compliant Triers” were characterized by a consistent trend of low probability of generating an MR alert as well as low probability of having controlled BP. “Compliant Achievers” were characterized by a consistent trend of low probability of generating MR alerts but high probability of having controlled BP. “Non-Compliant Achievers” were characterized by consistent trend of high probability of generating MR alert as well as high probability of having controlled BP.

**Table 3.** Latent class membership and estimated probabilities of each observed variable (N=491).

Variable^a^	Latent class 1: Compliant triers, Marginal mean^b^ (95% CI) (n=56, 11.4%)	Latent class 2: Compliant achievers, Marginal mean^b^ (95% CI) (n=368, 74.9%)	Latent class 3: Non-Compliant achievers, Marginal mean^b^ (95% CI) (n=67, 13.6%)
Missed Reading alert			
M7	0.20 (0.11‐0.34)	0.04 (0.02‐0.07)	0.72 (0.58‐0.83)
M8	0.06 (0.02‐0.19)	0.06 (0.04‐0.09)	0.65 (0.51‐0.77)
M9	0.11 (0.05‐0.24)	0.04 (0.02‐0.07)	0.78 (0.63‐0.88)
M10	0.09 (0.03‐0.22)	0.04 (0.02‐0.07)	0.76 (0.61‐0.86)
M11	0.14 (0.07‐0.29)	0.05 (0.03‐0.08)	0.70 (0.56‐0.81)
M12	0.15 (0.06‐0.30)	0.06 (0.04‐0.10)	0.70 (0.56‐0.81)
Blood pressure control^c^			
M7	0.44 (0.30‐0.59)	0.97 (0.94‐0.98)	0.74 (0.39‐0.93)
M8	0.38 (0.23‐0.55)	0.98 (0.95‐0.99)	0.82 (0.56‐0.94)
M9	0.55 (0.41‐0.69)	0.99 (0.97‐1.00)	0.71 (0.41‐0.90)
M10	0.49 (0.34‐0.64)	0.98 (0.96‐0.99)	0.81 (0.53‐0.94)
M11	0.48 (0.33‐0.62)	1.00 (0.00‐1.00)	0.76 (0.49‐0.91)
M12	0.40 (0.26‐0.56)	0.98 (0.95‐0.99)	0.87 (0.59‐0.97)

a M7 to M12 denote Month 7 to Month 12.

b Marginal mean presented here can have a value between 0 to 1 with values close to 1 indicating a higher probability of the observed variable with the latent class.

c BP Control is defined and categorized as yes if the monthly average systolic BP is less than 140 mm Hg and the monthly average diastolic BP is less than 90 mm Hg.

**Table 4.** Latent class size and key characteristics (N=491).

Class number and name	Size in sample (N=491), n (%)	Marginal probability (95% CI)	Key characteristics
Latent Class 1: Compliant Triers	56 (11.4)	0.12 (0.09-0.17)	Consistent trend of low probability of generating an MR^a^ alert and low probability of having controlled BP^b^
Latent Class 2: Compliant Achievers	368 (74.9)	0.74 (0.70-0.79)	Consistent trend of low probability of generating an MR alert and high probability of having controlled BP
Latent Class 3: Non-Compliant Achievers	67 (13.6)	0.13 (0.10-0.17)	Consistent trend of high probability of generating an MR alert and high probability of having controlled BP

a MR: Missing Reading.

b BP: blood pressure.

The most prevalent latent class identified in the current sample of participants was latent class 2 or “Compliant Achievers” with 74.9% (368/491) of the total sample compared to the other 2 latent classes (ie, latent class 1 or “Compliant Triers” and latent class 3 or “Non-Compliant Achievers”). Descriptive characteristics of identified latent classes are presented in Table 5.

**Table 5.** Descriptive characteristics of identified latent classes (N=491).

Characteristics	Compliant triers (n=56, 11.4%)	Compliant achievers (n=368, 74.9%)	Non-Compliant achievers (n=67, 13.6%)	Total (N=491)	*P* value
Age (years), mean (SD)	58.1 (9.8)	61.8 (10.0)	59.5 (10.4)	61.0 (10.1)	.02
Age (years), n (%)					.07
65 years or less	41 (73.2)	218 (59.2)	46 (68.7)	305 (62.1)	
More than 65 years	15 (26.8)	150 (40.8)	21 (31.3)	186 (37.9)	
Gender, n (%)					.72
Female	21 (37.5)	153 (41.6)	30 (44.8)	204 (41.6)	
Male	35 (62.5)	215 (58.4)	37 (55.2)	287 (58.5)	
Implementation site, n (%)					.21
A	16 (28.6)	75 (20.4)	16 (23.9)	107 (21.8)	
B	27 (48.2)	237 (64.4)	39 (58.2)	303 (61.7)	
C	13 (23.2)	56 (15.2)	12 (17.9)	81 (16.5)	
Baseline BP control**^a^**, n (%)					.02
No	43 (76.8)	209 (56.9)	37 (55.2)	289 (59.0)	
Yes	13 (23.2)	158 (43.1)	30 (44.8)	201 (41)	
Duration on PTEC-HT^b^ Program in days**^c^**, mean (SD)	471.6 (69.3)	471.6 (70.6)	493.6 (73.9)	474.6 (71.1)	.06
MR^d^ alert in index month, n (%)					<.001
No	48 (85.7)	330 (89.7)	20 (29.9)	398 (81.1)	
Yes	8 (14.3)	38 (10.3)	47 (70.1)	93 (18.9)	

a Baseline BP control is defined based on the first blood pressure reading submitted at the point of recruitment. A patient participant is categorized as having controlled baseline BP if they have both systolic BP <140 mm Hg and diastolic BP <90 mm Hg.

b PTEC-HT: Primary Technology Enhanced Care for Hypertension Program.

c Duration on PTEC-HT Program is computed based on the time between the recruitment date for a patient participant and the start of the index month during which the MR alert generator status was confirmed.

d MR: Missed Reading.

The 3 latent classes had significant differences for age, baseline BP control as well as MR alert generation in the index month. The average age of “Compliant Achievers” (mean 61.8, SD 10 years) was significantly higher than “Compliant Triers” (mean 58.1, SD 9.8 years) and “Non-Compliant Achievers” (mean 59.5, SD 10.4 years) (*P*=.02). Both “Compliant Achievers” (43.1%, 158/368) and “Non-Compliant Achievers” (44.8%, 30/67) had similar proportions of participants with controlled baseline BP at recruitment, and this was higher as compared to “Compliant Triers” (23.2%, 13/56) (*P*=.02). “Non-Compliant Achievers” had the highest proportion of MR alert generators during the index month (70.1%, 47/67) as compared to “Compliant Achievers” (10.3%, 38/368) and “Compliant Triers” (14.3%, 8/56) (*P*<.001).

**Figure 2.**
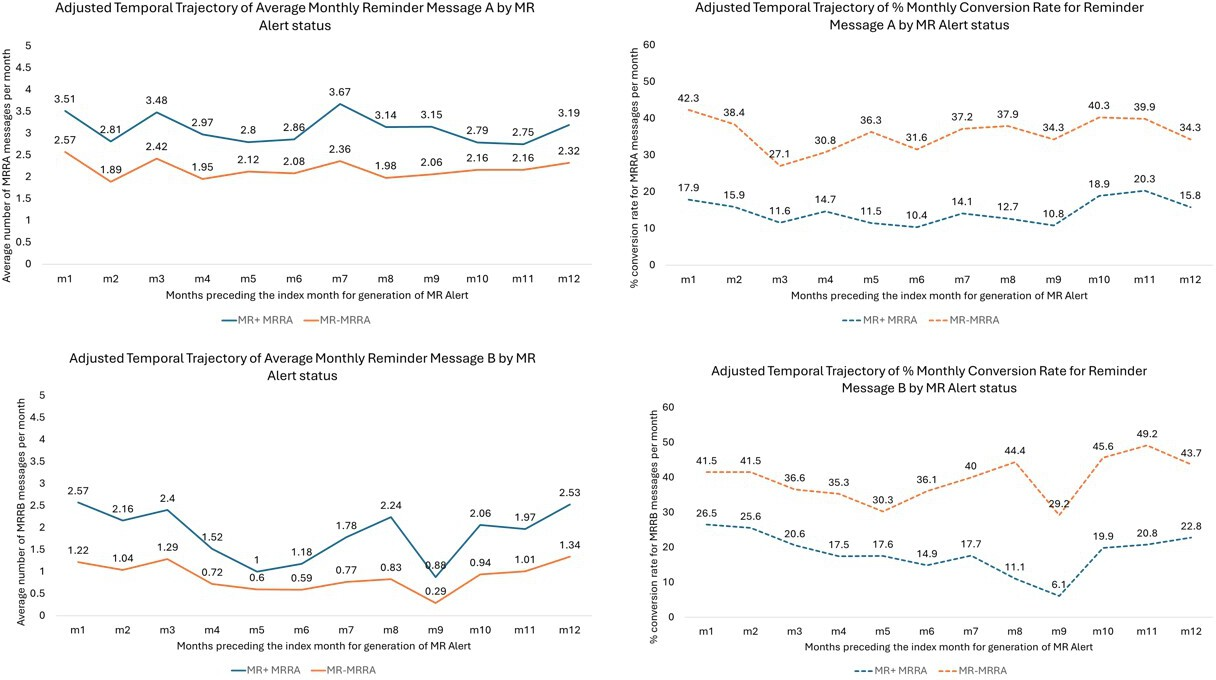
Adjusted temporal trajectories of Missed Reading Reminder A and Missed Reading Reminder B message counts and associated conversion rates stratified by Missed Reading alert generation status. MRRA: Missed Reading Reminder A; MRRB: Missed Reading Reminder B.

The findings for describing temporal trajectories of MRRA messages, conversion rate to MRRA messages, MRRB messages, and conversion rate to MRRB messages are presented in MultimediaAppendices 3^6^. Based on adjusted estimates from Model III, participants had a statistically significant consistent trajectory of generating MRRA messages ranging from 2.15 per month to 2.82 per month over 12 months preceding the index month of generation of the MR alert (*P*<.001) (refer to Multimedia Appendix 3). In addition, participants had a statistically significant consistent trajectory for conversion to MRRA messages with a conversion rate ranging from 23% to 36% per month over the 12 months preceding the index month of generation of the MR alert (*P*<.001) (refer to Multimedia Appendix 4). Based on adjusted estimates from Model III, participants had a statistically significant consistent trajectory of generating MRRB messages ranging from 0.51 to 1.91 per month over 12 months preceding the index month of generation of the MR alert (*P*<.001) (refer to Multimedia Appendix 5). In addition, participants had a statistically significant consistent trajectory for conversion to MRRB messages with a conversion rate ranging from 20% to 40% per month over 12 months preceding the index month of generation of the MR alert (*P*<.001) (refer to Multimedia Appendix 6). The interaction term between time and MR alert generation in the index month was statistically significant for all 4 temporal trajectories analyses. The adjusted temporal trajectories stratified by MR alert generation status (in index month) are presented for MRRA message monthly counts, MRRA message monthly conversion rate, MRRB message monthly counts, and MRRB monthly conversion rate in Figure 2.

In summary, MR alert generators have consistently higher MRRA message and MRRB message monthly counts and consistently lower monthly conversion rates to both types of messages as compared to non-MR alert generators over 12 months preceding the index month of generation of the MR alert.

## Discussion

### Principal Findings

With the overall aim of assessing user behavior, user profiles, generation of MR Alerts and associated factors in long-term users of PTEC-HT in public primary care settings in Singapore, our analysis of 491 participants from PTEC-HT revealed significant differences between MR alert generators and nongenerators. Age and program duration were significantly associated with the generation of the MR alert, with younger participants more likely to generate alerts and a monthly increase in duration associated with an 11% higher MR alert probability. Three latent classes were identified: (1) “Compliant Triers” (with low MR alerts and poor BP control), (2) “Compliant Achievers” (with low MR alerts and good BP control), and (3) “Non-Compliant Achievers” (with high MR alerts and good BP control). “Compliant Achievers” were the most prevalent group at 74.9%. Temporal trajectory analysis showed consistently higher MRRA and MRRB message counts among MR alert generators, but lower conversion rates compared to nongenerators. These insights suggest distinct participant profiles with differing BP control patterns and alert generation tendencies, highlighting potential areas for targeted interventions to improve MR alert management and BP outcomes. We are the first to the best of our knowledge to report such comprehensive findings on long-term user behavior and user profiles within a nationwide implemented technology-enabled intervention for self-monitoring of BP in patients with hypertension.

The results of our study align with and contribute to the growing body of evidence on the relationship between demographic characteristics, program duration, and telehealth intervention customization in the context of MR alert generation. Our findings indicated that younger participants were more likely to generate MR alerts, while older participants demonstrated more consistent adherence and lower alert generation. This aligns with results reported previously [18]. Similarly, another study found that older adults were less likely to enroll but participated equally and stayed longer than younger adults [19]. In addition, a recent study found younger age to be the strongest predictor of lower compliance in an online cardiac rehabilitation program [20]. We found that longer participation in PTEC-HT was linked to more MR alerts, aligning with evidence that telehealth compliance declines over time. Factors supporting better compliance include health literacy, implementation strategy, user training, and level of human support [21]. Another study similarly found that intermittent telehealth device use predicted earlier dropout from the program [22]. Thus, optimizing program duration requires balancing patient needs, funding, and subsidies to sustain engagement and manage MR alerts effectively.

LCA of the PTEC-HT program revealed 3 distinct participant profiles: “Compliant Triers,” “Compliant Achievers,” and “Non-Compliant Achievers.” Compliant Triers are characterized by high adherence to home BP monitoring and frequent engagement with the app, yet only modest improvements in BP control. Compliant Achievers similarly demonstrate diligent self-monitoring but, in contrast, attain significant BP reductions with diminishing alert frequency, reflecting successful engagement that translates into clinical gains. By comparison, Non-Compliant Achievers show minimal engagement with the app in terms of submitting their BP readings while still maintaining controlled BP, suggesting that some patients reach targets with limited digital support. Thus, our results revealed distinct latent classes with differing MR alert generation patterns, suggesting that customizing program features based on these user profiles may optimize compliance and outcomes. For example, Compliant Triers may require intensified coaching or treatment adjustments to convert their efforts into better BP control, Compliant Achievers should be reinforced to sustain their effective self-management practices, and even Non-Compliant Achievers could benefit from periodic re-engagement and follow-up to ensure long-term success. Seto et al [23] demonstrated the effectiveness of telemonitoring systems tailored to individual patients, highlighting the role of real-time feedback and ease of use in promoting adherence. Similarly, another study emphasized the need for flexibility and regular human interaction in a tele-exercise program [24]. Notably, the most common profile, Latent Class 2 or “Compliant Achievers,” represented 74.9% of the cohort, suggesting that concurrent compliance is associated with better BP control. While this study focused on concurrent patterns, future research could build on these findings by conducting a temporal analysis to evaluate whether compliance with BP reading submission predicts sustained BP control over longer periods, such as after 12 months.

Differences in MRRA and MRRB message patterns between MR alert generators and nongenerators highlight the need for ongoing compliance monitoring, as early interaction trends can predict long-term adherence and help identify participants at risk of disengagement [25]. This was supported by a recent systematic review associating early telehealth compliance with lower dropout rates among patients with chronic conditions [26]. Our analysis showed that MR alert generators had significantly more MRRA and MRRB messages but lower conversion rates, indicating reduced responsiveness despite higher messaging. This aligns with prior findings that the effectiveness of reminder messages tends to diminish over time, possibly due to alert fatigue or declining attention [27]. While our study did not explore the reasons for this decline, we have identified this as an important area for future qualitative research. Understanding patient perspectives could help explain declining engagement and guide targeted improvements. In the meantime, interventions may benefit from dynamic text message content, diversified communication channels, and periodic re-engagement strategies to sustain long-term responsiveness.

The following are several actionable recommendations based on our findings. Younger participants, who are more likely to generate MR alerts, would benefit from proactive engagement through educational sessions and digital tools. As program duration increases, additional support such as regular follow-ups and reminders can mitigate the rising trend of MR alerts. Tailored interventions based on latent class membership are essential: “Compliant Triers” need BP control support (eg, up-titration of antihypertensive medication), “Compliant Achievers” require sustained engagement, and “Non-Compliant Achievers” would benefit from adherence-focused strategies. Moreover, improving MR message conversion rates through simplified content and educational efforts is crucial, given consistently high message generation but low conversion. Finally, regular monitoring of message trajectories can support early intervention, ultimately enhancing BP control and program effectiveness.

The following are the strengths of our study. This study used data from the PTEC-HT scaling program, a nationwide telehealth initiative implemented across all public primary care clinics in Singapore. The use of real-world, population-level data enhances the external validity of the findings, making the results more applicable to similar large-scale telehealth interventions. The longitudinal design enabled the tracking of MR alert generation, BP control, and message interactions over a 12-month period. This approach allowed for the identification of temporal trends and patterns, such as the consistent decline in message conversion rates and the relationship between program duration and MR alert generation. By analyzing MR alert generation patterns in conjunction with BP control status, participant compliance, and telehealth message interactions, the study generated meaningful insights into user behavior. This granular level of analysis supports the potential for personalized telehealth interventions based on user profiles.

Our study has several limitations that warrant consideration. While we included a range of demographic, clinical, and engagement-related variables, we did not capture psychosocial factors such as patient motivation, technology literacy, or perceptions of telehealth interventions, which are factors known to influence adherence and engagement with digital health tools. This limitation is inherent in studies relying on IT system usage data and clinical outcomes without the inclusion of self-reported participant data. In addition, we did not perform a formal sample size calculation, as the study used data from all eligible participants. Furthermore, the analysis did not adjust for external factors such as policy changes, participant life events, or evolving health care practices, which may have influenced telehealth engagement. We acknowledge the potential impact of these unmeasured confounders on the observed relationships between MR alert generation, BP control, and text message interaction patterns, though the feasibility of collecting such contextual data was limited within this study framework. Finally, as the study was conducted in Singapore’s public primary care setting, the findings may not be fully generalizable to health care systems with different telehealth infrastructures, patient demographics, or care delivery models.

### Conclusion

This study successfully met its objectives by quantitatively characterizing the subgroup of PTEC-HT participants who generated MR alerts, identifying key factors associated with MR alert generation, and exploring distinct participant profiles. Younger age and longer program duration were significantly linked to MR alert generation, with LCA identifying 3 participant profiles differing in BP control and alert patterns. MR alert generators consistently showed higher MRRA and MRRB message counts but lower conversion rates over the 12 months before the index month. These findings highlight the need to tailor interventions by user profile, monitor compliance trends to detect disengagement early, and refine messaging to boost responsiveness. Future studies should examine psychosocial and behavioral drivers of engagement and strategies to sustain it over time, offering valuable insights to strengthen PTEC-HT’s role in hypertension care.

## Supplementary material

10.2196/74051 Multimedia Appendix 1 Operationalization of time points of data extraction from the PTEC-HT system.

10.2196/74051 Multimedia Appendix 2 Model fit indices table.

10.2196/74051 Multimedia Appendix 3 Temporal trajectory of Missed Reading Reminder A messages over 12 months preceding the index month of generation of Missed Reminder Alert.

10.2196/74051 Multimedia Appendix 4 Temporal trajectory of conversion rate to Missed Reading Reminder A messages over 12 months preceding the index month of generation of Missed Reading Alert.

10.2196/74051 Multimedia Appendix 5 Temporal trajectory of Missed Reading Reminder B messages over 12 months preceding the index month of generation of Missed Reminder Alert.

10.2196/74051 Multimedia Appendix 6 Temporal trajectory of conversion rate to Missed Reading Reminder B messages over 12 months preceding the index month of generation of Missed Reading Alert.

## Abbreviations

AIC: Akaike information criteria
BIC: Bayesian information criterion
BP: blood pressure
GEE: generalized estimating equation
LCA: latent class analysis
MOHT: Ministry of Health Office for Healthcare Transformation
MR: Missed Reading
MRRA: Missed Reading Reminder A
MRRB: Missed Reading Reminder B
OR: odds ratio
PTEC-HT: Primary Technology Enhanced Care for Hypertension Program
