# Supplementary material for: Exploring User Behavior, Profiles, and Generation of Missed Reading Alerts in Long-Term Users of a Technology-Enabled Intervention for Self-Monitoring of Blood Pressure in Public Primary Care Setting in Singapore: Longitudinal Observational Study
Source: J Med Internet Res. 2025 Sep 22;27:e74051. doi: 10.2196/74051 (PMC12453572; doi:10.2196/74051)
Supplement: Multimedia Appendix 1 [file jmir-v27-e74051-s001.docx]

**Supplementary Table 1**. Operationalization of time-points of data extraction from PTEC-HT system^a^

| **Recruitment Period** | **Observation Period** | **Contributing Period** | **Index Period** |
| --- | --- | --- | --- |
| A patient having recruitment or enrolment date over this period would be eligible to be included in this analysis. | 12 months prior to the contributing period. This period is defined to describe the profiles of MR alert generators as well as temporal trajectories of receiving missed reading reminder messages and associated conversion rates. | The period over which the patient did not submit a weekly reading continuously for 4 weeks. | The period over which the main outcome of interest, (i.e., MR alert on clinician dashboard) appears. |
| Anytime Jun 2022 or before | Jul 2022 to Jun 2023 | Jul 2023 | Aug 2023 |
| ^a^Note for data extraction from PTEC-HT system: 1. MR alert generator Group: Condition of MR alert in Aug (*Yes*) AND (2) Recruitment anytime Jun 22 or before; 2. non-MR alert generator Group: Condition of MR alert in Aug (*No*) AND (2) Recruitment anytime Jun 22 or before | | | |
