# Supplementary material for: Exploring User Behavior, Profiles, and Generation of Missed Reading Alerts in Long-Term Users of a Technology-Enabled Intervention for Self-Monitoring of Blood Pressure in Public Primary Care Setting in Singapore: Longitudinal Observational Study
Source: J Med Internet Res. 2025 Sep 22;27:e74051. doi: 10.2196/74051 (PMC12453572; doi:10.2196/74051)
Supplement: Multimedia Appendix 2 [file jmir-v27-e74051-s002.docx]

**Supplementary Table 2**. Model Fit Indices Table

|  | 2-class Model  (base^a^) | 3-class Model  (base^a^) | 3-class Model  (base^a^ + BP control^b^) | 3-class Model  (base^a^ + compliance^c^) | 3-class Model  (base^a^ + BP control^b^+ compliance^c^) |
| --- | --- | --- | --- | --- | --- |
| AIC | 1977.443 | 1939.4204 | 3197.0268 | 4486.9313 | 5954.3222 |
| BIC | 2031.9967 | 2023.3493 | 3356.4916 | 4646.3962 | 6189.3231 |
| ^a^Base model comprises of the MR alert variable in preceding 6 months.  ^b^BP control is categorised as yes if the monthly average systolic BP is less than 140 mm Hg AND monthly average diastolic BP is less than 90 mm Hg.  ^c^Compliance is defined as monthly average of submitted BP reading of 4 or more. | | | | | |
