# Supplementary material for: Exploring User Behavior, Profiles, and Generation of Missed Reading Alerts in Long-Term Users of a Technology-Enabled Intervention for Self-Monitoring of Blood Pressure in Public Primary Care Setting in Singapore: Longitudinal Observational Study
Source: J Med Internet Res. 2025 Sep 22;27:e74051. doi: 10.2196/74051 (PMC12453572; doi:10.2196/74051)
Supplement: Multimedia Appendix 3 [file jmir-v27-e74051-s003.docx]

**Supplementary Table 3**. Temporal trajectory of **Missed Reading Reminder A (MRRA)** messages over 12 months preceding the index month of generation of MR Alert

|  |  | **Model 1** |  | **Model 2** |  | **Model 3** |  |
| --- | --- | --- | --- | --- | --- | --- | --- |
|  |  | **Margin (95% CI)** | **P value** | **Margin (95% CI)** | **P value** | **Margin (95% CI)** | **P value** |
| **Time** | **Month1** | 2.78 (2.63-2.94) | <.001 | 2.79 (2.64-2.95) | <.001 | 2.82 (2.68-2.97) | <.001 |
|  | **Month2** | 2.10 (1.98-2.21) |  | 2.11 (1.99-2.23) |  | 2.15 (2.04-2.26) |  |
|  | **Month3** | 2.66 (2.52-2.81) |  | 2.67 (2.52-2.81) |  | 2.70 (2.56-2.84) |  |
|  | **Month4** | 2.19 (2.07-2.31) |  | 2.19 (2.07-2.31) |  | 2.23 (2.12-2.35) |  |
|  | **Month5** | 2.27 (2.15-2.39) |  | 2.27 (2.16-2.39) |  | 2.32 (2.20-2.43) |  |
|  | **Month6** | 2.25 (2.13-2.37) |  | 2.26 (2.14-2.38) |  | 2.31 (2.19-2.43) |  |
|  | **Month7** | 2.68 (2.53-2.82) |  | 2.68 (2.54-2.83) |  | 2.73 (2.60-2.87) |  |
|  | **Month8** | 2.26 (2.14-2.37) |  | 2.26 (2.14-2.38) |  | 2.30 (2.20-2.41) |  |
|  | **Month9** | 2.32 (2.21-2.43) |  | 2.32 (2.21-2.44) |  | 2.37 (2.26-2.47) |  |
|  | **Month10** | 2.30 (2.18-2.42) |  | 2.31 (2.19-2.43) |  | 2.34 (2.22-2.45) |  |
|  | **Month11** | 2.28 (2.16-2.40) |  | 2.29 (2.17-2.41) |  | 2.33 (2.21-2.45) |  |
|  | **Month12** | 2.52 (2.36-2.68) |  | 2.53 (2.37-2.69) |  | 2.58 (2.42-2.73) |  |
| **MR Alert in index month** | |  |  |  |  |  |  |
|  | **No** |  |  |  |  | 2.20 (2.11-2.28) | <.001 |
|  | **Yes** |  |  |  |  | 3.14 (2.97-3.32) |  |
| Model A: time variable (12 months preceding the index month of generation of MR Alert)  Model B: Model A + age, gender, cluster, baseline BP control, duration of PTEC-HT programme  Model C: Model B + MR Alert (during index month)  Model D: Model C + interaction term (i.e., time variable*MR Alert) | | | | | | | |
