# Supplementary material for: Exploring User Behavior, Profiles, and Generation of Missed Reading Alerts in Long-Term Users of a Technology-Enabled Intervention for Self-Monitoring of Blood Pressure in Public Primary Care Setting in Singapore: Longitudinal Observational Study
Source: J Med Internet Res. 2025 Sep 22;27:e74051. doi: 10.2196/74051 (PMC12453572; doi:10.2196/74051)
Supplement: Multimedia Appendix 4 [file jmir-v27-e74051-s004.docx]

**Supplementary Table 4**. Temporal trajectory of **conversion rate to Missed Reading Reminder A (MRRA)** messages over 12 months preceding the index month of generation of MR Alert

|  |  | **Model 1** |  | **Model 2** |  | **Model 3** |  |
| --- | --- | --- | --- | --- | --- | --- | --- |
|  |  | **Margin (95% CI)** | **P value** | **Margin (95% CI)** | **P value** | **Margin (95% CI)** | **P value** |
| **Time** | **Month1** | 0.36 (0.32-0.41) | <.001 | 0.36 (0.32-0.40) | <.001 | 0.36 (0.32-0.40) | <.001 |
|  | **Month2** | 0.33 (0.29-0.37) |  | 0.33 (0.29-0.37) |  | 0.32 (0.28-0.36) |  |
|  | **Month3** | 0.24 (0.20-0.27) |  | 0.23 (0.20-0.27) |  | 0.23 (0.20-0.26) |  |
|  | **Month4** | 0.27 (0.23-0.31) |  | 0.27 (0.23-0.31) |  | 0.26 (0.23-0.30) |  |
|  | **Month5** | 0.30 (0.26-0.34) |  | 0.30 (0.26-0.34) |  | 0.30 (0.26-0.34) |  |
|  | **Month6** | 0.27 (0.23-0.31) |  | 0.27 (0.23-0.31) |  | 0.26 (0.22-0.30) |  |
|  | **Month7** | 0.32 (0.28-0.36) |  | 0.32 (0.28-0.36) |  | 0.31 (0.27-0.35) |  |
|  | **Month8** | 0.32 (0.28-0 .36) |  | 0.32 (0.28-0.36) |  | 0.31 (0.27-0.35) |  |
|  | **Month9** | 0.29 (0.25-0.33) |  | 0.29 (0.25-0.33) |  | 0.28 (0.24-0.32) |  |
|  | **Month10** | 0.35 (0.31-0.39) |  | 0.35 (0.31-0.39) |  | 0.35 (0.31-0.39) |  |
|  | **Month11** | 0.35 (0.31-0.40) |  | 0.35 (0.31-0.40) |  | 0.35 (0.31-0.39) |  |
|  | **Month12** | 0.30 (0.26-0.34) |  | 0.30 (0.26-0.34) |  | 0.29 (0.25-0.33) |  |
| **MR Alert in index month** | |  |  |  |  |  |  |
|  | **No** |  |  |  |  | 0.36 (0.33-0.38) | <.001 |
|  | **Yes** |  |  |  |  | 0.14 (0.10-0.18) |  |
| Model A: time variable (12 months preceding the index month of generation of MR Alert)  Model B: Model A + age, gender, cluster, baseline BP control, duration of PTEC-HT programme  Model C: Model B + MR Alert (during index month)  Model D: Model C + interaction term (i.e., time variable*MR Alert) | | | | | | | |
