# Supplementary material for: Exploring User Behavior, Profiles, and Generation of Missed Reading Alerts in Long-Term Users of a Technology-Enabled Intervention for Self-Monitoring of Blood Pressure in Public Primary Care Setting in Singapore: Longitudinal Observational Study
Source: J Med Internet Res. 2025 Sep 22;27:e74051. doi: 10.2196/74051 (PMC12453572; doi:10.2196/74051)
Supplement: Multimedia Appendix 5 [file jmir-v27-e74051-s005.docx]

**Supplementary Table 5**. Temporal trajectory of **Missed Reading Reminder B (MRRB)** messages over 12 months preceding the index month of generation of MR Alert

|  |  | **Model 1** |  | **Model 2** |  | **Model 3** |  |
| --- | --- | --- | --- | --- | --- | --- | --- |
|  |  | **Margin (95% CI)** | **P value** | **Margin (95% CI)** | **P value** | **Margin (95% CI)** | **P value** |
| **Time** | **Month1** | 1.76 (1.59-1.93) | <.001 | 1.76 (1.59-1.92) | <.001 | 1.79 (1.64-1.95) | <.001 |
|  | **Month2** | 1.49 (1.34-1.63) |  | 1.48 (1.33-1.63) |  | 1.54 (1.40-1.68) |  |
|  | **Month3** | 1.77 (1.62-1.92) |  | 1.76 (1.61-1.91) |  | 1.79 (1.64-1.94) |  |
|  | **Month4** | 1.03 (0.91-1.15) |  | 1.03 (0.91-1.15) |  | 1.07 (0.94-1.19) |  |
|  | **Month5** | 0.78 (0.67-0.89) |  | 0.78 (0.67-0.89) |  | 0.81 (0.69-0.92) |  |
|  | **Month6** | 0.82 (0.70-0.95) |  | 0.82 (0.69-0.94) |  | 0.86 (0.73-0.98) |  |
|  | **Month7** | 1.15 (1.04-1.27) |  | 1.15 (1.03-1.26) |  | 1.20 (1.09-1.30) |  |
|  | **Month8** | 1.33 (1.18-1.47) |  | 1.32 (1.18-1.46) |  | 1.36 (1.23-1.50) |  |
|  | **Month9** | 0.49 (0.39-0.58) |  | 0.48 (0.38-0.58) |  | 0.51 (0.41-0.61) |  |
|  | **Month10** | 1.37 (1.23-1.51) |  | 1.37 (1.24-1.51) |  | 1.40 (1.27-1.53) |  |
|  | **Month11** | 1.41 (1.27-1.55) |  | 1.40 (1.27-1.54) |  | 1.44 (1.31-1.58) |  |
|  | **Month12** | 1.85 (1.67-2.04) |  | 1.85 (1.67-2.03) |  | 1.91 (1.73-2.09) |  |
| **MR Alert in index month** | |  |  |  |  |  |  |
|  | **No** |  |  |  |  | 1.05 (0.96-1.14) | <.001 |
|  | **Yes** |  |  |  |  | 2.06 (1.84-2.28) |  |
| Model A: time variable (12 months preceding the index month of generation of MR Alert)  Model B: Model A + age, gender, cluster, baseline BP control, duration of PTEC-HT programme  Model C: Model B + MR Alert (during index month)  Model D: Model C + interaction term (i.e., time variable*MR Alert) | | | | | | | |
