# Supplementary material for: Exploring User Behavior, Profiles, and Generation of Missed Reading Alerts in Long-Term Users of a Technology-Enabled Intervention for Self-Monitoring of Blood Pressure in Public Primary Care Setting in Singapore: Longitudinal Observational Study
Source: J Med Internet Res. 2025 Sep 22;27:e74051. doi: 10.2196/74051 (PMC12453572; doi:10.2196/74051)
Supplement: Multimedia Appendix 6 [file jmir-v27-e74051-s006.docx]

**Supplementary Table 6**. Temporal trajectory of **conversion rate to Missed Reading Reminder B (MRRB)** messages over 12 months preceding the index month of generation of MR Alert

|  |  | **Model 1** |  | **Model 2** |  | **Model 3** |  |
| --- | --- | --- | --- | --- | --- | --- | --- |
|  |  | **Margin (95% CI)** | **P value** | **Margin (95% CI)** | **P value** | **Margin (95% CI)** | **P value** |
| **Time** | **Month1** | 0.38 (0.33-0.43) | <.001 | 0.37 (0.32-0.42) | <.001 | 0.36 (0.31-0.41) | <.001 |
|  | **Month2** | 0.37 (0.32-0.43) |  | 0.37 (0.32-0.42) |  | 0.36 (0.31-0.41) |  |
|  | **Month3** | 0.33 (0.28-0.37) |  | 0.33 (0.28-0.37) |  | 0.32 (0.27-0 .36) |  |
|  | **Month4** | 0.31 (0.25-0.36) |  | 0.31 (0.25-0.36) |  | 0.29 (0.24-0.34) |  |
|  | **Month5** | 0.27 (0.22-0.33) |  | 0.27 (0.22-0.33) |  | 0.26 (0.20-0 .31) |  |
|  | **Month6** | 0.31 (0.25-0.36) |  | 0.31 (0.25-0.36) |  | 0.29 (0.23-0.34) |  |
|  | **Month7** | 0.33 (0.28-0.39) |  | 0.33 (0.28-0.39) |  | 0.32 (0.27-0.37) |  |
|  | **Month8** | 0.35 (0.29-0.40) |  | 0.35 (0.29-0.40) |  | 0.33 (0.28-0.38) |  |
|  | **Month9** | 0.22 (0.17-0.27) |  | 0.23 (0.17-0 .28) |  | 0.20 (0.15-0.25) |  |
|  | **Month10** | 0.38 (0.33-0.44) |  | 0.38 (0.33-0.43) |  | 0.37 (0.32-0.42) |  |
|  | **Month11** | 0.41 (0.36-0.47) |  | 0.41 (0.36-0.47) |  | 0.40 (0.35-0.45) |  |
|  | **Month12** | 0.38 (0.33-0.43) |  | 0.38 (0.33-0.43) |  | 0.36 (0.32-0.41) |  |
| **MR Alert in index month** | |  |  |  |  |  |  |
|  | **No** |  |  |  |  | 0.39 (0.36-0 .43) | <.001 |
|  | **Yes** |  |  |  |  | 0.19 (0.15-0 .23) |  |
| Model A: time variable (12 months preceding the index month of generation of MR Alert)  Model B: Model A+ age, gender, cluster, baseline BP control, duration of PTEC-HT programme  Model C: Model B + MR Alert (during index month)  Model D: Model C + interaction term (i.e., time variable*MR Alert) | | | | | | | |
